# Toll-Like Receptor 9 Signaling Delays Neutrophil Apoptosis by Increasing Transcription of Mcl-1

**DOI:** 10.1371/journal.pone.0087006

**Published:** 2014-01-22

**Authors:** Driss El Kebir, Anas Damlaj, János G. Filep

**Affiliations:** Research Center, Maisonneuve-Rosemont Hospital and Department of Pathology and Cell Biology, University of Montréal, Montréal, Quebec, Canada; Harvard Medical School, United States of America

## Abstract

Neutrophils detect bacterial constituents, including bacterial DNA (CpG DNA), which elicits innate immunity and prolongs the functional life span of neutrophils through suppression of apoptosis. Both the anti-apoptotic protein Mcl-1 and activation of NF-κB have been implicated in neutrophil survival, but there is no evidence that these are linked in neutrophils. We hypothesized that CpG DNA could simultaneously activate these pathways. High purity CpG DNA (0.4–3.2 µg/ml) extended the life span of human neutrophils in vitro by delaying apoptosis through altering the rate of Mcl-1 turnover. CpG DNA slightly decreased Mcl-1 protein level in the presence of cyclohexmide and the proteasome inhibitor MG132 had little effect on Mcl-1 expression in CpG DNA-treated neutrophils. In contrast, CpG DNA evoked rapid increases in DNA binding by NF-κB/p65 and Mcl-1 mRNA. NF-κB inhibitors and the telomere-derived TLR9 inhibitory oligonucleotide 5′-TTT AGG GTT AGG GTT AGG G-3′ markedly reduced Mcl-1 protein levels and subsequently abrogated suppression of apoptosis by CpG DNA. Furthermore, CpG DNA attenuated the decreases in Mcl-1 in both cell lysate and nucleus of neutrophils undergoing spontaneous apoptosis and increased Mcl-1 translocation to the mitochondria, leading to preservation of mitochondrial transmembrane potential. These results demonstrate that CpG DNA through toll-like receptor 9 links two survival signaling pathways by delaying apoptosis through induction of NF-κB-mediated Mcl-1 gene transcription and promoting Mcl-1 translocation to the mitochondria.

## Introduction

Neutrophils are essential effectors of innate immune response to infection and tissue injury. Circulating neutrophils have the shortest lifespan among leukocytes and are functionally quiescent [1]. Neutrophil trafficking into inflamed tissues is associated with extended survival through delaying constitutive apoptosis, which allows performing their immune functions effectively. Excessive neutrophil responses or impaired neutrophil clearance contribute to persisting tissue damage that underlies many inflammatory diseases [2]. Neutrophil survival/apoptosis emerged as one of the control points that ultimately determine the outcome of the inflammatory response [3]. Thus, suppression of neutrophil apoptosis results in aggravation and prolongation of tissue injury in various models of inflammation [4]–[6]. Delayed neutrophil apoptosis is also apparent in patients with inflammatory diseases, including acute respiratory distress syndrome [7], acute coronary syndromes [8] and sepsis [9], [10].

Bacterial genomic DNA contains short sequences of unmethylated CpG dinucleotides (CpG DNA) that are recognized by toll-like receptor-9 (TLR9) [11], also expressed by neutrophils [12], [13] and other DNA sensing molecules [14]. CpG DNA activates neutrophils [15], promotes neutrophil trafficking into the primary sites of infection [16]–[20] and suppresses neutrophil apoptosis *in vitro* [13], [21]. CpG DNA has been detected in the lung of cystic fibrosis patients [17], coronary artery specimens [22], and in the blood of critically ill patients who have had negative blood culture [23], pathologies that are associated with delayed neutrophil apoptosis [7]–[10].

Neutrophil apoptosis is regulated by a complex network of signalling pathways. A characteristic feature of neutrophil apoptosis is the pre-eminence of the Bcl-2 homolog Mcl-1 as a key survival protein [24], [25]. Mcl-1 has a high turnover rate [26], which is well suited for dynamic control of neutrophil survival. Mcl-1 expression inversely correlates with the degree of neutrophil apoptosis *in vitro* [27], in experimental endotoxemia [28] and in patients with Crohn’s disease [29]. Mcl-1 appears to be an upstream trigger of apoptosis and a downstream target of caspase activity in human neutrophils [30]. Survival signals, such as GM-CSF, preserve Mcl-1 expression predominantly through inhibition of its proteasomal degradation [31]. Importantly, therapeutic strategies to induce neutrophil apoptosis with cyclin-dependent kinase inhibitors [32], aspirin-triggered 15-epi-lipoxin A4 [33], annexin A1 [34] and resolvin E1 [35] appear to be mediated through modulation of Mcl-1 expression. Suppression of apoptosis in neutrophils in whole blood by TLR agonists, including CpG DNA was found to coincide with higher intracellular Mcl-1 levels [21], however, whether this could be attributed to a direct action of CpG DNA on neutrophils as well as the underlying molecular mechanisms have not been elucidated. Here we report that CpG DNA delays apoptosis of human neutrophils by enhancing Mcl-1 expression predominantly through TLR9 and NF-κB-mediated induction of Mcl-1 gene transcription and Mcl-1 translocation to the mitochondria.

## Materials and Methods

### Ethics Statement

The Clinical Research Committee at the Maisonneuve-Rosemont Hospital (Montréal, Québec, Canada) had approved all protocols (reference number: 99097) and we obtained written consent from each blood donor.

### Bacterial DNA

*Escherichia coli* DNA (strain B) (Sigma-Aldrich, Mississauga, Ontario, Canada) was purified by extraction with phenol: chloroform: isoamyl alcohol (25∶21:1, vol/vol/vol) and ethanol precipitation [16]. DNA preparations contained <5 ng LPS/mg DNA by *Limulus assay.* Ultra pure LPS (*E. coli*, O111:B4 strain) and endotoxin-free DNA from *E. coli* (K12-DNA, endotoxin <0.06 EU/µg DNA) were obtained from InvivoGen (San Diego, CA, USA).

### Neutrophil Isolation and Culture

Freshly isolated neutrophils were obtained [5] from venous blood (anticoagulated with sodium heparin, 50 U/ml) of healthy volunteers who had denied taking any medication for >2 weeks. Neutrophils (5×10^6^ cells/ml, purity >96%, viability >98%, apoptotic <2%) were resuspended in Hanks’ balanced salt solution supplemented with 10% autologous serum. Neutrophils were cultured on a rotator for 20 min at 37°C with the human TLR9 inhibitor phosphorothioate oligodeoxynucleotide 5′-ttt agg gtt agg gtt agg gttv agg g-3′ (iODN, 0.6 or 2.4 µM, InvivoGen) [36], a negative control ODN 5′-tgc tgc tgc ttg caa gca gct tga t-3′(ctrl-ODN, 2.4 µM, InvivoGen), cycloheximide (10 µg/ml), MG132 (10 µM, Sigma-Aldrich) or the selective NF-κB inhibitors SN50 (4 µM, Calbiochem-EMD Biosciences, La Jolla, CA, USA) or BAY 11–7082 (10 µM, Calbiochem) and then challenged with CpG DNA (0.025–6.4 µg/ml). Previous studies have shown that maximum suppression of neutrophil apoptosis by CpG DNA was achieved at 6.4 µg/ml [13] and the CpG DNA concentrations used in this study are similar to those detected in the sputum of patients with cystic fibrosis [17]. At the indicated times, cells were processed as described below.

### Assessment of Apoptosis and Mitochondrial Transmembrane Potential (ΔΨ_m_)

Apoptosis was assessed with flow cytometry using FITC-conjugated annexin-V (BD Biosciences) in combination with propidium iodide (Molecular Probes, Eugene, OR, USA), and the percent of cells with hypoploid DNA [5]. To monitor ΔΨ_m_, neutrophils (5×10^5^ cells) were incubated for 30 min with the lipophilic fluorochrome chloromethyl-X-rosamine (CMXRos, 200 nM, Molecular Probes) and the fluorescence was analyzed in a FACSCalibur flow cytometer and CellQuestPro software (BD Biosciences, Mountain View, CA, USA) [5].

### Mcl-1 Protein Expression

Neutrophils were lysed in 1x Laemmli buffer containing 1∶100 (vol/vol) protease inhibitor cocktail set (Thermo Scientific, Nepean, Ontario, Canada). Nuclear and mitochondrial factions were prepared with the NE-PER Cytoplasmic Extraction kit and the Mitochondria Isolation kit respectively (both from Thermo Scientific). Proteins from 10^7^ neutrophils, nuclear or mitochondrial factions were resolved by SDS-PAGE, transferred to Immun-Blot™-PVDF membrane (Bio-Rad Laboratories, Mississauga, Ontario, Canada), blocked with 5% nonfat milk, and probed with antibodies to Mcl-1 (rabbit polyclonal Ab, sc- 819, Santa Cruz Biotechnology, Santa Cruz, CA, USA), YY1 (rabbit polyclonal Ab, sc-1703, Santa Cruz Biotechnology), superoxide dismutase-2 (SOD-2, mouse mAb, clone A-2, Santa Cruz Biotechnology) or β-actin (Sigma-Aldrich) (5). Band density was quantified using the National Institutes of Health ImageJ software (http://rsb.info.nih.gov/ij/) and was expressed as a ratio of unstimulated cells following correction for loading discrepancies using the density of the actin, YY1 or SOD-2 band as appropriate.

### Detection of Mitochondrion-associated Mcl-1

To assess mitochondria-associated Mcl-1, neutrophils were first stained with MitoTracker Red (Molecular Probes) for 30 min at 37°C, then seeded onto L-polylysine-coated coverslips, fixed in 4% formaldehyde for 15 min, and permeabilized with 0.2% Triton X-100 in TBS for 15 min. After washing with PBS, cells were blocked with 2% bovine serum albumin in PBS for 2 hours, incubated with rabbit anti-Mcl-1 mAb (Santa Cruz Biotechnology) or an irrelevant class-matched Ab overnight, followed by staining with FITC-labeled goat anti-rabbit Ab (Jackson ImmunoResearch Laboratories, West Grove, PA, USA). Images were captured in a LSM700 confocal laser scanning microscope (Carl Zeiss, Jena, Germany). Percentage of Mcl-1-positive cells (defined as staining above a threshold level set by staining the cells with an irrelevant class-matched Ab plus the secondary Ab) was assessed by an observer unaware of the treatments. Mitochondrial localisation of Mcl-1 was assessed in 10 randomly selected neutrophils per sample that stained positive for Mcl-1 by calculating the Pearson’s coefficient using Image J software.

### Mcl-1 Gene Expression

Total RNA (1 µg) extracted with TRIzol reagent (Invitrogen, Carlsbad, CA, USA) was reverse transcribed into cDNA using Superscript III reverse transcriptase (Invitrogen). Quantitative real-time PCR was performed on an ABI 7500 Sequence Detection System (Applied Biosystems, Burlington, Ontario, Canada) using Platinum SYBR Green Super Mix (Invitrogen). The following primers were used: Mcl-1 forward, GGACATCAAAAACGAAGACG, reverse, GCAGCTTTCTTGGTTTATGG; 18s rRNA forward, GCAATTATTCCCCATGAACG, and reverse GGCCTCACTAAACCATCCAA. Mcl-1 values were normalized using 18s rRNA as an endogenous control.

### NF-κB Activation

Nuclear fractions were prepared with the NE-PER Nuclear and Cytoplasmic Extraction kit (Thermo Scientific). Binding of NF-κB/p65 to the immobilized κB consensus sequence 5′-GGGACTTTCC-3′ was assayed with the TransAM NF-κB/p65 Activation Assay (Active Motif, Carlsbad, CA, USA) using 15 µg nuclear extracts. Binding is expressed as optical density (OD) following correction with binding in the presence of 20 pmol wild-type consensus oligonucleotide. A mutated consensus oligonucleotide served as a negative control.

### TLR9 Expression

To visualize cytoplasmic location of TLR9 and CpG DNA, neutrophils were first incubated for 5 min with Alexa Fluor 488-labeled CpG DNA prepared with the ULYSIS Alexa Fluor 488 Nucleic Acid Labeling kit (Molecular Probes), then seeded onto L-polylysine-coated coverslips, fixed in 4% formaldehyde for 15 min, and permeabilized with Permeabilization Buffer (eBioscience, San Diego, CA, USA). After washing with PBS, cells were blocked with 1% bovine serum albumin in PBS for 1 hour, incubated with R-PE-conjugated anti-TLR9 Ab eB72-1665 or an irrelevant class-matched Ab (eBioscience) overnight and then counterstained with the nuclear stain 4′6-diaminidino-2-phenylindole (DAPI). Images were captured in a Leica DMRI fluorescence microscope equipped with a digital camera (Retiga EX, QImaging, Surrey, BC, Canada) and OpenLab software (OpenLab Srl, Florence, Italy).

### Statistical Analysis

Data are expressed as mean ± SEM. Statistical comparisons were made by ANOVA using ranks followed by Dunn’s multiple contrast hypothesis tests, the Wilcoxon signed rank test or by the Mann-Whitney U test (two-tailed). P values <0.05 were considered statistically significant.

## Results

### CpG DNA Delays Neutrophil Apoptosis through TLR9

**Figure 1:**
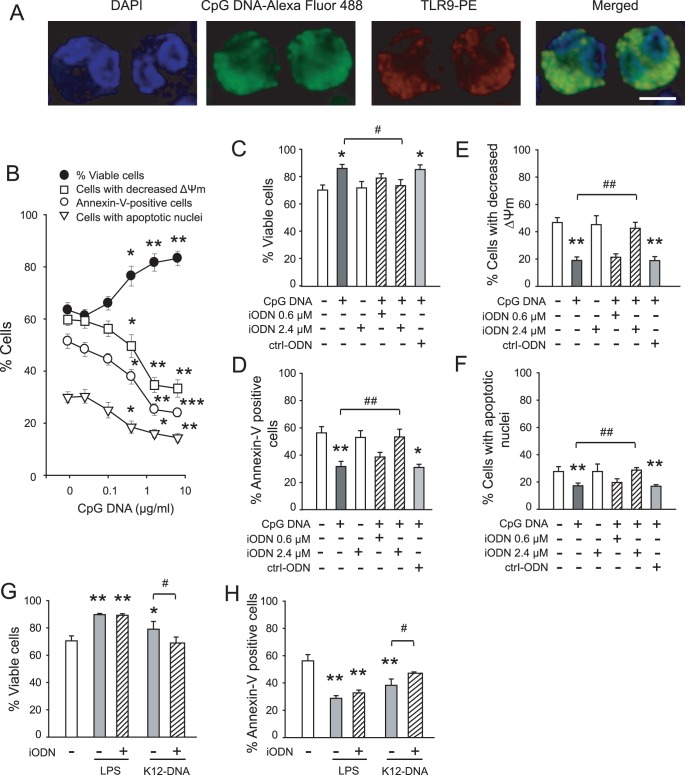
Bacterial DNA delays apoptosis of human neutrophils through TLR9. (**A**) Co-localisation of CpG DNA and TLR9. Neutrophils were incubated with Alexa Fluor 488-labeled CpG DNA and then stained with R-PE-conjugated anti-TLR9 eB72-1665 and 4′,6-diamidino-2-phenylindole (DAPI). Images were captured using a Leica DMRI fluorescence microscope and are representative of 3 neutrophil preparations from different blood donors. Scale bar: 10 µm. (**B**) Concentration-dependence of the effects of CpG DNA on neutrophil apoptosis. Neutrophils (5×10^6^ cells/ml) were cultured for 24 h with increasing concentrations of CpG DNA and viability (propidium iodide staining), mitochondrial transmembrane potential (ΔΨ_m_) (CMXRos staining) and apoptosis (annexin-V-FITC binding and nuclear DNA content) were assessed. (**C–F**) Telomere-derived iODN inhibits the actions of CpG DNA. Neutrophils were incubated for 10 min with iODN (0.6 or 2.4 µM) or ctlr-ODN (2.4 µM) and then challenged with CpG DNA (1.6 µg/ml) for 24 h. (**G, H**) K12-DNA delays neutrophil apoptosis. Neutrophils were cultured for 24 h with LPS-free K-12-DNA (1.6 µg/ml) or ultra pure LPS (1 µg/ml) and viability (**G**) and annexin-V-FITC binding (**H**) were assessed. Data are means ±SEM (n = 3–5). *P<0.05; **P<0.01; ***P<0.001 vs. untreated.^ #^P<0.05; ^##^P<0.01.

We used immunofluorescence microscopy to visualize TLR9 expression in human neutrophils and confirmed earlier reports [12], [13] that human neutrophils express high levels of TLR9 (Figure 1A). Following culture of neutrophils with Alexa Fluor 488-labeled CpG DNA, TLR9 staining coincided with intracellular Alexa Fluor 488-conjugated CpG DNA (Figure 1A). Consistent with published data [13], CpG DNA suppressed constitutive apoptosis (assessed by positive staining for annexin-V, decreased mitochondrial transmembrane potential and nuclear DNA content) and increased neutrophil viability in a concentration-dependent manner (Figure 1B). Pretreatment of neutrophils with the telomere-derived iODN, which selectively blocks CpG DNA binding to TLR9 [36], almost completely prevented the apoptosis suppressing action of CpG DNA (Figure 1C–F). iODN alone had no detectable effects on neutrophil viability (Figure 1C) or apoptosis (Figure 1D–F) and did not affect the apoptosis suppressing action of ultra pure LPS (Figure 1G, H). Ctrl-ODN failed to affect neutrophil responses to CpG DNA (Figure 1C–F). Furthermore, iODN also prevented the effects K12-DNA, a commercial high purity *E. coli* DNA preparation (Figure 1G, H).

### CpG DNA Induces Transcription of Mcl-1 through Activation of NF-κB

**Figure 2:**
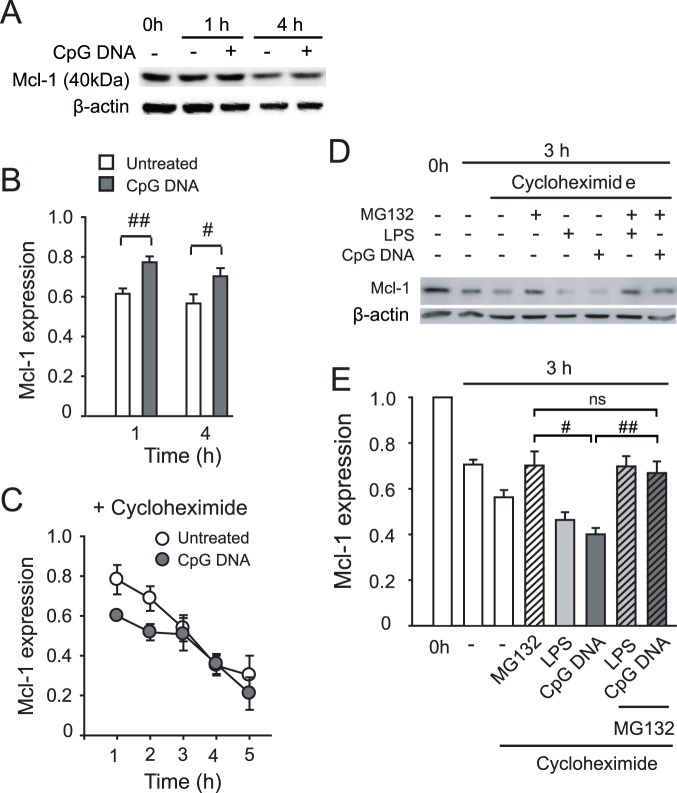
CpG DNA preserves Mcl-1 expression in neutrophils. Neutrophils (5×10^6^ cells/ml) were cultured with CpG DNA (1.6 µg/ml) in the presence of cycloheximide (10 µg/ml) or MG132 (10 µM) for the indicated times, lysed and Mcl-1 expression was determined by western blot analysis. Mcl-1 expression is expressed as band density relative to untreated, freshly isolated neutrophils (0 h) following correction with density of the corresponding actin band. (**A, B**) Time course of Mcl-1 expression in the absence (**A,** representative blot, **B** densitometry analysis) and presence of cycloheximide (**C**). Data are means ±SEM (n = 4–7). (**D, E**) Mcl-1 expression in the presence of MG132± cycloheximide. Neutrophils were cultured with CpG DNA for 3 hours. (**D**) Representative blot and (**E**) densitometry analysis from 4–5 independent experiments. Data are means ±SEM. ns, not significant, ^#^P<0.05; ^##^P<0.01.

As anticipated, in the presence of CpG DNA Mcl-1 protein quantities fell much more slowly than in untreated neutrophils (Figure 2A and B depict a representative blot and densitometry analyses, respectively). In cycloheximide-treated neutrophils, CpG DNA accelerated Mcl-1 degradation at 1 and 2 h of culture and slightly shortened the half-life of Mcl-1 (Figure 2C), however, these changes did not reach statistical significance (calculated Mcl-1 half-life was 139±17 vs. 121±24 min in the absence and presence of CpG DNA, respectively, n = 6, P>0.05). As anticipated, the proteasome inhibitor MG132 preserved Mcl-1 expression (Figure 2D and E depict a representative blot and densitometry analyses, respectively). CpG DNA evoked further decreases in Mcl-1 in cycloheximide-treated neutrophils that was reversed by MG132 (Figure 2D and E), indicating that CpG DNA does not regulate neutrophil apoptosis by preventing the proteasomal degradation of Mcl-1.

**Figure 3:**
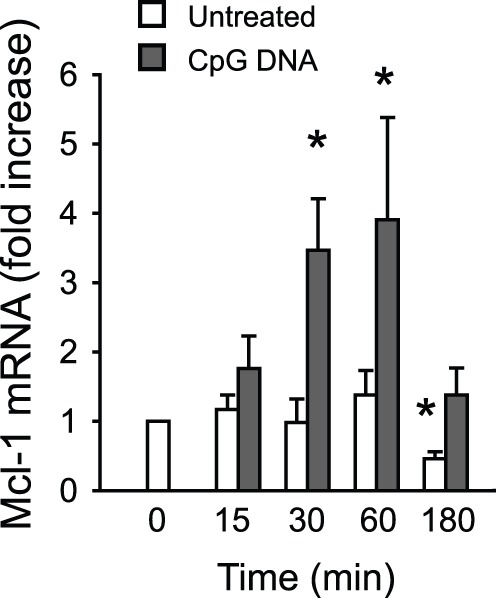
CpG DNA induces Mcl-1 gene transcription. Neutrophils (10^7^ cells/ml) ± CpG DNA (1.6 µg/ml) were incubated for the indicated times. Mcl-1 gene expression was assessed by quantitative real-time PCR and was normalized using 18s rRNA as an endogenous control. Data are means ±SEM (n = 3–5). *P<0.05 vs. untreated at time 0.

We next investigated whether CpG DNA modulates Mcl-1 expression at the level of transcription. CpG DNA induced rapid increases in Mcl-1 mRNA with a 3.9-fold increase observed at 60 min (Figure 3). The effects of CpG DNA were still detectable at 3 hours of culture, when Mcl-1 mRNA expression in untreated neutrophils dropped below 50% of that detected in freshly isolated neutrophils (Figure 3).

**Figure 4:**
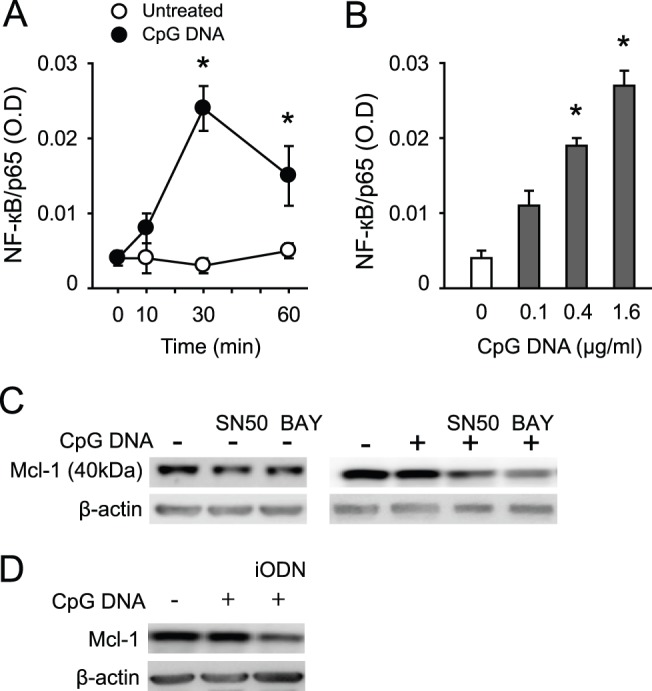
CpG DNA activates NF-κB in human neutrophils. (**A, B**) Time and concentration-dependent activation of NF-κB. Neutrophils (5×10^6^ cells/ml) were cultured with CpG DNA (1.6 µg/ml) for the indicated times (**A**) or with different concentrations of CpG DNA for 30 min (**B**). Nuclear extracts were prepared and DNA binding of NF-κB was detected by ELISA using an immobilized κB consensus sequence and expressed as OD following corrections with binding in the presence of 20 pmol wild-type consensus oligonucleotide. A mutated consensus oligonucleotide did not affect binding. Data are means ±SEM (n = 4). *P<0.05 vs. untreated at time 0. Inhibition of Mcl-1 protein expression by selective NF-κB inhibitors (**C**) and iODN (**D**). Neutrophils were incubated with CpG DNA (1.6 µg/ml) in the presence of SN 50 (4 µM), BAY 11-7082 (BAY, 10 µM) or iODN (2.4 µM) for 30 min, and Mcl-1 expression was assessed by western blot analysis. Blots are representative of 3 experiments with different blood donors.

Because ligation of TLR9 triggers NF-κB activation [37] and the Mcl-1 gene promoter contains the κB binding motif [25], we prepared neutrophil nuclear fractions and assessed NF-κB/p65 binding to immobilized κB consensus sequence with ELISAs. CpG DNA evoked rapid increases in DNA binding by NF-κB/p65, peaking at 30 min post-CpG DNA (Figure 4A). This action was concentration-dependent (Figure 4B). Pretreatment of neutrophils with the selective NF-κB inhibitors SN50 or BAY 11-7082 markedly reduced Mcl-1 protein expression in neutrophils challenged with CpG DNA (Figure 4C). Likewise, iODN produced similar reductions in Mcl-1 expression (Figure 4D).

### CpG DNA Modulation of Intracellular Localization of Mcl-1

**Figure 5:**
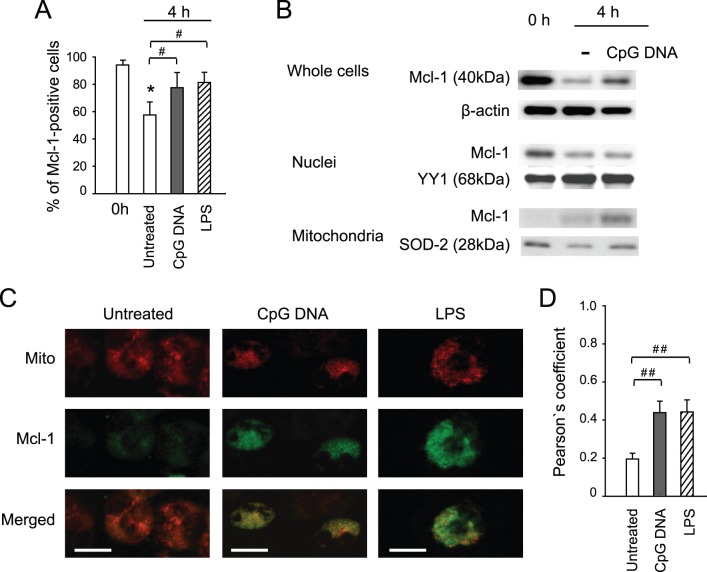
CpG DNA affects intracellular localization of Mcl-1. Freshly isolated neutrophils (0 h) or neutrophils (10^7^ cells/ml) cultured with CpG DNA (1.6 µg/ml) or LPS (1 µg/ml) for 4 h were studied. (**A**) Mcl-1-positive cells. Neutrophils were stained with a polyclonal rabbit anti-Mcl-1 Ab followed by a goat anti-rabbit FITC-conjugated Ab. (**B**) Intracellular localization of Mcl-1. Whole cell lysates, nuclear and mitochondrial fractions were prepared and analysed for Mcl-1. β-actin, YY1 and SOD-2 served as loading controls, respectively. Results are representative of 3 independent experiments with different blood donors. (**C**) CpG DNA induces Mcl-1 translocation to mitochondria. Following 4 h of culture, neutrophils were first stained with MitoTracker Red (Mito), then with a rabbit anti-Mcl-1 Ab followed by a goat anti-rabbit FITC-conjugated Ab. Staining with an irrelevant rabbit Ab served as a negative control. Images were captured in a Zeiss LSM700 confocal laser scanning microscope. Results are representative of 5 neutrophil preparations from different donors. Scale bars: 10 µm. (**D**) Mitochondrial localisation of Mcl-1 was assessed in neutrophils (10 randomly selected cells per sample) that stained positive for Mcl-1 by calculating the Pearson’s coefficient using Image J software. Data are means ±SEM (n = 5–8). *P<0.05 vs. freshly isolated neutrophils (0 h). ^#^P<0.05;^ ##^P<0.01.

As anticipated, almost all freshly isolated neutrophils stained positive for Mcl-1 and the percentage of Mcl-1-positive neutrophils fell rapidly during the first 4 hours of culture (Figure 5A). In the presence of CpG DNA considerably higher percentage of neutrophils stained positive for Mcl-1 and the effect of CpG DNA was comparable to that of high purity LPS (Figure 5A). To address intracellular distribution of Mcl-1, whole cell lysates, nuclear and mitochondrial fractions were prepared and subjected to western blot analysis. In freshly isolated neutrophils, Mcl-1 was detectable in whole cell lysates and nuclear fractions, but not in the mitochondria (Figure 5B). Culture of untreated neutrophils for 4 hours resulted in decreases in Mcl-1 protein level in whole cell lysates and nuclear fractions, whereas Mcl-1 became detectable in the mitochondrial fraction (Figure 5B). CpG DNA attenuated the decreases in Mcl-1 in whole cell lysates and nuclear fractions, and enhanced Mcl-1 level in the mitochondrial fraction (Figure 5B). To confirm these latter observations, we monitored mitochondrial localization of Mcl-1 by confocal microscopy. Figure 5C shows representative images. Analysis of Pearson’s coefficients revealed a marked increase in Mcl-1 and mitochondria association in neutrophils challenged with CpG DNA as compared with untreated neutrophils (Figure 5D). These actions of CpG DNA were comparable to those of high purity LPS (Figure 5C, D).

## Discussion

Earlier and the present studies have demonstrated that CpG DNA at pathologically relevant concentrations [17] delays neutrophil apoptosis [13], [21], which likely contributes to aggravation and/or prolongation of the inflammatory response. Our present findings provide several insights into the underlying mechanisms and signal transduction pathways. Our data support a model wherein CpG DNA signals through TLR9 to suppress apoptosis via induction of NF-κB-mediated transcription of the Mcl-1 gene and subsequent translocation of Mcl-1 protein to mitochondria.

To date, TLR9 remains the only known receptor for immunostimulatory DNA [11], [37] Accumulating evidence indicates that CpG DNA predominantly signals through this receptor to delay neutrophil apoptosis. Indeed, human neutrophils express TLR9 intracellularly [12], [13] and we found that CpG DNA colocalizes with this receptor. Inhibition of endosomal acidification, a critical step to initiate signaling through TLR9 from the endosome [11], [38], [39] rendered the apoptotic machinery unresponsive to CpG DNA [13]. Methylating cytosines in CpG dinucleotides in bacterial DNA resulted in an almost complete loss of its apoptosis delaying action [13]. The present results with iODN lend further support to the notion that CpG DNA binding to TLR9 is required to confer its functions in neutrophils. This iODN contains the potent TLR9 inhibitory sequence TTAGGG multimers found in mammalian telomeres, diffuses into cells and disrupts interaction of CpG DNA with TLR9 in endosomal vesicles without affecting cellular uptake of CpG DNA [36]. Furthermore, iODN produced similar degree of inhibition of CpG DNA action as previously observed with endosomal acidification inhibitors [13]. Recent results suggest that TLR9-independent cytosolic DNA sensors, such as DAI (DNA-dependent activator of IFN regulatory factors or DLM-1/ZBP1) or AIM-2 can activate innate immunity to endogenous DNA that escaped lysosomal degradation [14], [40]. However, the expression and function of such sensors in human neutrophils remains to be investigated.

The Bcl-2 family members, Mcl-1 and, to a lesser extent, A1 have been identified as key regulators of neutrophil apoptosis and survival [24]–[27]. Mcl-1 protein expression inversely correlates with the degree of neutrophil apoptosis in both experimental and clinical settings. Increased levels of Mcl-1 have been implicated in prolonging neutrophil survival by proinflammatory cytokines such as GM-CSF, IL-1 and TNF [27], [31]. We extended these observations to CpG DNA, and identified a mechanism distinct from those activated by cytokines. Unlike other members of the Bcl-2 family, Mcl-1 has a very short half-life [26], [31] and its cellular level changes substantially as neutrophils age and upon exposure of neutrophils to inflammatory mediators [26]. These changes can occur by changes in its rate of turnover or by modulation of transcription or translation of the Mcl-1 gene. Previous studies have identified increased Mcl-1 stability rather than enhanced transcription as a key event for GM-CSF signaling of neutrophil survival [26], [31], [41]. GM-CSF induces ERK-mediated phosphorylation of Thr^163^ within the PEST domain, thus protecting Mcl-1 from proteolysis [42]. Mcl-1 contains PEST sequences and other motifs that target it for proteasomal degradation [25], [43], and the proteasome appears to be a major route by which Mcl-1 undergoes degradation following polyubiquitination by the E3 ubiquitin ligase Mule/ARF-BP1 during constitutive apoptosis [44]. Consistently, proteasome inhibitors can block Mcl-1 turnover as well as execution of the constitutive death program [31], [42]. We detected further decreases in Mcl-1 expression by CpG DNA in cycloheximide-treated neutrophils, which were prevented in the presence of MG132, indicating that unlike GM-CSF, CpG DNA does not prevent the proteasomal degradation of Mcl-1. In contrast, CpG DNA markedly enhanced transcription of Mcl-1 in human neutrophils, and this effect was detectable as early as 15 min post-CpG DNA. At 3-h culture, Mcl-1 mRNA levels in CpG DNA-treated neutrophils were comparable to those in freshly isolated neutrophils, when untreated neutrophils exhibited markedly reduced Mcl-1 mRNA levels. These observations indicate that enhanced *Mcl-1* transcription accounted for most variation of Mcl-1 protein expression in CpG DNA-treated neutrophils. Our findings also demonstrate that CpG DNA can maintain Mcl-1 expression to delay apoptosis in isolated neutrophils as assessed at 24 h of culture. By contrast, suppression of neutrophil apoptosis in whole blood requires 30–100-fold higher concentrations of CpG DNA than in isolated neutrophils and critically depends on cytokine production by monocytes and other leukocytes after 4 hours of culture [21]. The promoter region of *Mcl-1* contains an array of putative and confirmed transcription factor binding sites, including NF-κB binding site (residues −218/−208), cAMP response elements and consensus STAT response elements [25]. NF-κB has been proposed to generate survival cues for neutrophils [45], [46], but curiously, there is no evidence for direct NF-κB regulation of *Mcl-1* transcription in neutrophils. Neutrophil responses to TNF-α depend on the presence or absence of NF-κB-controlled survival proteins with a short half-life [45], which may indirectly regulate *Mcl-1*. Recent results suggest that inhibition of the phosphorylation of RNA polymerase II, a general regulator of gene transcription rather than direct inhibition of NF-κB is responsible for cyclin-dependent kinase inhibitor-driven downregulation of Mcl-1 and neutrophil apoptosis [47]. Furthermore, GM-CSF does not appear to signal through NF-κB [48]. We document for the first time that CpG DNA signals through NF-κB to enhance *Mcl-1* transcription. Following CpG DNA binding to TLR9, MyD88 associates with IRAK4, IRF5 and TRAF6, culminating in the activation of the canonical NF-κB pathway and MAP kinase pathway [reviewed in 37]. We detected rapid nuclear accumulation and DNA binding of NF-κB/p65 in neutrophils in response to CpG DNA. Pharmacological blockade of NF-κB activation markedly, although never completely, inhibited CpG DNA-stimulated expression of Mcl-1, indicating involvement of transcription factors other than NF-κB. Inhibition of CpG DNA-evoked Mcl-1 expression by iODN or NF-κB inhibitors lends additional support for the TLR9/NF-κB-mediated signaling for Mcl-1 gene transcription. Interestingly, the predominance of transcriptional regulation of *Mcl-1* by CpG DNA resembles those observed in cancer cells upon ER stress [49].

Our results also demonstrate CpG DNA induction of translocation of Mcl-1 from the cytoplasm to the mitochondrion. In freshly isolated neutrophils, Mcl-1 is predominantly localized to the cytoplasm and nucleus [25], [41] and this was confirmed in the present study. We could not detect Mcl-1 in the mitochondrial fraction by immunoblotting. Nuclear and whole cell lysate Mcl-1 rapidly decreases, whereas Mcl-1 in the mitochondrial fraction becomes detectable in neutrophils undergoing spontaneous apoptosis [50]. The mitochondria forms clusters to which the pro-apoptotic Bax is also localized [50], [51], resulting in decreases in mitochondrial transmembrane potential with subsequent release of cytochrome C and other pro-apoptotic proteins [50]. We have previously shown that signals generated via ERK and PI3K/Akt by CpG DNA are important to induce dissociation of Mcl-1 from its heterodimers with Bad or Bax in the cytoplasm [13]. This would permit targeting Mcl-1 to the mitochondria, where it can counter the activity of Bax, Bak and/or Bim, resulting in preservation of mitochondrial transmembrane potential and consequently prevention of cytochrome C release [13].

In the present study, we did not address the pathophysiological consequences of CpG DNA suppression of neutrophil apoptosis. CpG DNA prolongation of the functional life span may contribute to enhanced neutrophil defenses against invading pathogens. On the other hand, suppression of neutrophil apoptosis could also aggravate the inflammatory response and impair the resolution of inflammation [17], [20]. Indeed, CpG DNA has been reported to persist in tissues and the circulation even in the absence of live bacteria, where it likely contribute to maintaining neutrophil-mediated inflammation, as has been shown in the lung of patients with cystic fibrosis [17] and experimental models of acute lung injury [52]. Of note, TLR9 can also sense mitochondrial DNA relased from damaged cells [53], likely influencing neutrophil function and life span.

In summary, our results show that bacterial DNA prolongs neutrophil survival by delaying apoptosis through TLR9-dependent NF-κB-mediated induction of Mcl-1 gene transcription, thereby decreasing the rate of Mcl-1 turnover and subsequent promotion of Mcl-1 translocation to the mitochondria. In the view of the importance of sustained Mcl-1 expression in neutrophils during inflammation, accelerating Mcl-1 turnover by telomere-derived TLR9 inhibitory oligonucleotide may have a therapeutic potential in an environment where bacterial DNA is abundantly present.
